# Comparison among random forest, logistic regression, and existing clinical risk scores for predicting outcomes in patients with atrial fibrillation: A report from the J‐RHYTHM registry

**DOI:** 10.1002/clc.23688

**Published:** 2021-07-28

**Authors:** Eiichi Watanabe, Shunsuke Noyama, Ken Kiyono, Hiroshi Inoue, Hirotsugu Atarashi, Ken Okumura, Takeshi Yamashita, Gregory Y. H. Lip, Eitaro Kodani, Hideki Origasa

**Affiliations:** ^1^ Division of Cardiology, Department of Internal Medicine Fujita Health University Bantane Hospital Aichi Japan; ^2^ Division of Bioengineering, Graduate School of Engineering Science Osaka University Osaka Japan; ^3^ Department of Internal Medicine Saiseikai Toyama Hospital Toyama Japan; ^4^ Department of Internal Medicine AOI Hachioji Hospital Tokyo Japan; ^5^ Department of Cardiovascular Medicine Saiseikai Kumamoto Hospital Kumamoto Japan; ^6^ Department of Cardiovascular Medicine The Cardiovascular Institute Tokyo Japan; ^7^ Liverpool Centre for Cardiovascular Science University of Liverpool and Liverpool Heart & Chest Hospital Liverpool UK; ^8^ Department of Cardiovascular Medicine Nippon Medical School, Tama‐Nagayama Hospital Tokyo Japan; ^9^ Division of Biostatistics and Clinical Epidemiology University of Toyama Graduate School of Medicine and Pharmaceutical Sciences Toyama Japan

**Keywords:** arrhythmia, bleeding, machine learning, mortality, stroke, thrombosis

## Abstract

**Background:**

Machine learning (ML) has emerged as a promising tool for risk stratification. However, few studies have applied ML to risk assessment of patients with atrial fibrillation (AF).

**Hypothesis:**

We aimed to compare the performance of random forest (RF), logistic regression (LR), and conventional risk schemes in predicting the outcomes of AF.

**Methods:**

We analyzed data from 7406 nonvalvular AF patients (median age 71 years, female 29.2%) enrolled in a nationwide AF registry (J‐RHYTHM Registry) and who were followed for 2 years. The endpoints were thromboembolisms, major bleeding, and all‐cause mortality. Models were generated from potential predictors using an RF model, stepwise LR model, and the thromboembolism (CHADS_2_ and CHA_2_DS_2_‐VASc) and major bleeding (HAS‐BLED, ORBIT, and ATRIA) scores.

**Results:**

For thromboembolisms, the C‐statistic of the RF model was significantly higher than that of the LR model (0.66 vs. 0.59, *p* = .03) or CHA_2_DS_2_‐VASc score (0.61, *p* < .01). For major bleeding, the C‐statistic of RF was comparable to the LR (0.69 vs. 0.66, *p* = .07) and outperformed the HAS‐BLED (0.61, *p* < .01) and ATRIA (0.62, *p* < .01) but not the ORBIT (0.67, *p* = .07). The C‐statistic of RF for all‐cause mortality was comparable to the LR (0.78 vs. 0.79, *p* = .21). The calibration plot for the RF model was more aligned with the observed events for major bleeding and all‐cause mortality.

**Conclusions:**

The RF model performed as well as or better than the LR model or existing clinical risk scores for predicting clinical outcomes of AF.

## 1 INTRODUCTION

Atrial fibrillation (AF) is the most common sustained arrhythmia seen in the elderly population and is associated with an increased risk of thromboembolisms, major bleeding, and mortality. ^1^ Treatment decisions for AF are often made by risk prediction models built using a regression analysis, but their accuracy is modest. ^2^, ^3^, ^4^, ^5^, ^6^ AF is a highly heterogeneous condition caused by various underlying disorders, and a simple risk score may limit the performance of the risk stratification. ^7^ Therefore, more accurate and personalized risk stratification approaches are required.

Machine learning (ML), the use of mathematical algorithms that address the higher dimensional, nonlinear relationships among many variables, is making significant progress.^8^, ^9^, ^10^ Promising tools for ML in cardiology include the improvement of the automated risk prediction and interpretation of medical imaging that can have a dramatic impact on the practice of cardiology. Currently, several studies have shown that ML outperforms the risk prediction as compared to the traditional logistic models. Mortazavi et al. showed an improved prediction of readmissions for worsening heart failure with ML models as compared to a logistic regression (LR) analysis.^11^ In another study using a large multicenter database, the ML model was more accurate in detecting clinical deterioration in the hospitalized patients than the traditional regression models.^12^ In a more recent study using patients admitted to the intensive care unit, Hyland et al. developed a new approach that provides early identification of patients at risk for circulatory failure with a much lower false‐alarm rate than conventional threshold‐based systems.^13^ However, contradictory results have also been reported.^14^, ^15^ While AF patients represent an important target population for whom adverse events need predicting, few studies have applied ML to the risk assessment in them. Therefore, the aim of this study was to compare the discrimination and calibration performance of an ML algorithm called the random forest (RF) model, against a stepwise LR model and several conventional score based risk predictors, to predict thromboembolisms, major bleeding, and all‐cause mortality, using a prospective nationwide registry of AF patients.^16^, ^17^, ^18^

## 2 METHODS

### 2.1 Patients

For this study, we used individual patient data from the J‐RHYTHM Registry.^16^, ^17^, ^18^ The J‐RHYTHM Registry is an observational, prospective cohort study that enrolled patients with AF between January and July of 2009 at 150 sites within Japan. In this post‐hoc study, after excluding patients with mitral stenosis or those who had undergone mechanical valve replacements (*n* = 410), the final cohort included 7406 patients. Warfarin was used as an oral anticoagulation therapy because no direct oral anticoagulants were available when this registry was carried out. The study protocol conformed to the 1975 Declaration of Helsinki and was approved by the Nippon Medical School institutional review board and review board at each enrolling center. All patients gave their written informed consent. The data that support the findings of this study are available from the corresponding author upon reasonable request.

### 2.2 Endpoints

The endpoint of thromboembolisms included ischemic strokes, transient ischemic attacks, and systemic embolisms. Major bleeding as the safety endpoint included intracranial hemorrhage, gastrointestinal bleeding, and other causes of bleeding requiring hospitalization. The all‐cause mortality was also tallied. The diagnostic criteria for each event have been described in research design papers.^16^, ^17^ The patients were followed for 2 years, or until an endpoint, whichever occurred first. All analyses of the rates of the endpoints were based on the first event during follow‐up. A local investigator ascertained the events, and members of the outcomes review committee adjudicated all outcomes.

### 2.3 Risk scores

The components of the CHADS_2_ ^2^ and CHA_2_DS_2_‐VASc scores^3^ for thromboembolisms and the HAS‐BLED,^4^ ORBIT,^5^ and ATRIA^6^ scores for major bleeding are shown in the Supplementary file (Appendix S1). In the CHA_2_DS_2_‐VASc scores, we modified the “V” criterion to include coronary artery disease only, because no data were available regarding peripheral artery disease and aortic plaque. The time in therapeutic range (TTR) was determined with the method of Rosendaal et al. ^19^ and a labile international normalized ratio (INR) was defined as TTR < 60%. For this determination, the target INR level was set at 1.6–2.6 for patients aged 70 years or older and at 2.0–3.0 for patients aged younger than 70 years, in keeping with Japanese guidelines for AF pharmacotherapy.^20^ We assessed the predictive accuracy of the CHADS_2_ and CHA_2_DS_2_‐VASc scores for thromboembolisms and all‐cause mortality ^21^ and the HAS‐BLED, ORBIT, and ATRIA scores for major bleeding.

### 2.4 Statistical analysis

The statistical analyses were performed with R project software (R foundation, Vienna, Austria). An RF analysis was performed using the Scikit‐learn open‐source ML library, version 0.21.2. In this study, we used an RF algorithm, which is a decision tree‐based ensemble learning method for the classification, regression, and clustering of the data.^22^ The RF analysis was composed of three steps: (1) missing values imputation, (2) classification model building, and (3) feature selection.

### 2.5 Missing values imputation

There were 10 variables for which we did not have data from every single patient. They were the height (13.8%), body weight (13.1%), hemoglobin (11.5%), platelet count (11.6%), creatinine (11.1%), total cholesterol (25.5%), total bilirubin (29.5%), aspartate aminotransferase (AST) (12.1%), alanine aminotransferase (ALT) (11.7%), and creatinine clearance (11.1%). In cases with missing data, categorical variables were replaced by the modes, and numerical variables were imputed with sequential regression multivariate imputation.^23^

### 2.6 Classification model building

The RF classifier was trained (80% of an overall cohort) and tested (20%) on the feature‐selected variables. After hyperparameter tuning and feature selection on the training data, the model was fit to the training data set. The predictive capacity of the models was estimated by the mean value and 95% confidence interval of the C‐statistic over 5‐fold cross‐validation. In this study, the RF model was fit using 1000 trees.

### 2.7 Feature Selection

We used 42 variables in this study (Supplementary file, Appendix S2). The feature selection on the training data was performed using a sequential forward floating selection (SFFS).^24^ The SFFS is a family of greedy search algorithms, which is used to select a subset of features that is suitable for model building.

### 2.8 Permutation importance

To provide a description of an individualized prediction made by the algorithm, we measured the permutation importance on a testing dataset.^22^, ^25^ The permutation importance was calculated by measuring how the performance of a classifier decreased when a single predictive variable was randomly shuffled. Because shuffling breaks the association between the variable and target clinical outcome, the resulting drop in performance of the classifier as measured by the area under the curve (AUC) was indicative of how much the classifier depended on the predictive variable.

### 2.9 Stepwise LR analysis

We used the logit link function of R for stepwise multivariable LR. The predictive capacity of the regression model was estimated via the mean value and 95% confidence interval for the C‐statistic over the 5‐fold cross‐validation iterations.

### 2.10 Model calibration

The performances of the RF and LR models were evaluated with calibration plots comparing the expected and actual event rates for the outcomes. The RF outputs were reconverted into posterior probabilities by fitting the sigmoid functions.^26^ The risk of the outcomes was calculated for each sub‐interval bounded by the quintiles. A calibration slope smaller than one indicated an overestimation of the event risks for that quintile. We also evaluated the relationship between the existing clinical risk scores and the event rate. The existing risk scores were presented as a continuous score or classified into three categories (low, intermediate, and high risk) based on previous literature.^2^, ^3^, ^4^, ^5^, ^6^ The high‐risk event rate cutoff value was defined as the maximum event rate (mean value of the highest quintile interval) in the calibration curve of the RF model. We calculated the net reclassification improvement (NRI) by the NRI index and 95% confidence interval to assess the added value of the LR model or risk scores compared to the RF model.^27^ A continuous NRI was used to compare the RF and LR, and a categorical NRI was used to compare the RF model and existing risk scores.^2^, ^3^, ^4^, ^5^, ^6^ The baseline variables are presented as the number and frequency or mean ± *SD* values, or the median and interquartile range. The DeLong test was used to compare the C‐statistics between the models.^28^ A two‐tailed *p* value of <.05 was considered significant.

## 3 RESULTS

The baseline characteristics of the patients are shown in Table 1. We analyzed 7406 patients with nonvalvular AF (age 69.8 ± 10.0 years, female 29.2%). A total of 6404 patients (86.5%) were taking warfarin. The prevalence of a previous stroke or transient ischemic attack, or major bleeding were 13.8% and 4.5%, respectively. Supplemental Table S2 shows the number of patients with the thromboembolism risk scores and major bleeding risk scores divided into three categories.

**TABLE 1:** Baseline characteristics of the patients

	Overall (*n* = 7406)
Age, years	69.8 ± 10.0
	71 [64–77]
Age ≥ 75 years	2565 (34.6)
Male, (*n*) %	5241 (70.8)
Height (cm)	162 ± 9.1
Weight (kg)	62.2 ± 12.2
Systolic blood pressure (mmHg)	126.0 ± 16.2
Diastolic blood pressure (mmHg)	73.5 ± 17.0
Heart rate (beat per min)	72.5 ± 13.2
Type of AF, *n* (%)	
Paroxysmal	2835 (38.3)
Persistent	1081 (14.6)
Permanent	3490 (47.1)
Comorbidities, *n* (%)	
Congestive heart failure	2055 (27.7)
Hypertension	4481 (60.5)
Diabetes	1359 (18.3)
Previous stroke or TIA	1022 (13.8)
Coronary artery disease	781 (10.5)
COPD	131 (1.8)
Malignancy	567 (7.7)
Cardiomyopathy	634 (8.6)
Congenital heart disease	96 (1.3)
Hyperthyroidism	131 (1.8)
Abnormal renal or liver function	901 (12.2)
Alcohol use >8 U/week	2263 (30.6)
Labile INR	3330 (44.9)
History of hepatitis	316 (4.3)
Previous bleeding, *n* (%)	
Intracranial	81 (1.1)
Gastrointestinal	170 (2.3)
Other sites	78 (1.1)

Laboratory data	
Hemoglobin (g/dl)	13.7 ± 1.7
Platelet (×10^4^/ul)	23.3 ± 25.7
Creatinine (mg/dl)	0.96 ± 0.56
CCr (ml/min)	68.5 ± 26.6
Total cholesterol (mg/dl)	188.8 ± 36.7
Total bilirubin (mg/dl)	0.82 ± 2.5
AST (IU/L)	26.1 ± 10.9
ALT (IU/L)	22.6 ± 13.1
Medications, *n* (%)	
Warfarin	6404 (86.5)
Antiplatelet agents	1937 (26.2)
Antihypertensive drugs	5354 (72.2)
Class I Antiarrhythmic drug	1248 (16.9)
Class III Antiarrhythmic drug	223 (3.0)
Beta‐blocker	753 (10.2)
Digitalis	622 (8.4)
CCB	270 (3.6)
ACE/ARB	3934 (53.1)
Statin	1795 (24.2)

*Note:* Data represent number, frequency, or means ± *SD*.

Abbreviations: Abnormal liver function, chronic hepatic disease or significant hepatic derangement (e. g., bilirubin >2× upper limit of normal, in association with aspartate aminotransferase/alanine aminotransferase/alkaline phosphatase >3× upper limit normal); Abnormal renal function, chronic dialysis, renal transplantation, or serum creatinine >2.26 mg/dl; ACE, angiotensin converting enzyme inhibitor; AF, atrial fibrillation; ALT, alanine aminotransferase; Antihypertensive drugs include α‐blocker, dihydropyridine calcium channel blocker, and diuretics; ARB, angiotensin II type 1 receptor blocker; AST, aspartate aminotransferase; CCB, nondihydropyridine calcium channel blocker; CCr, creatinine clearance; COPD, chronic obstructive pulmonary disease; INR, international normalized ratio of prothrombin time; labile INR, therapeutic time in range < 60%; TIA, transient ischemic attack.

### 3.1 Model performance

**FIGURE 1:**
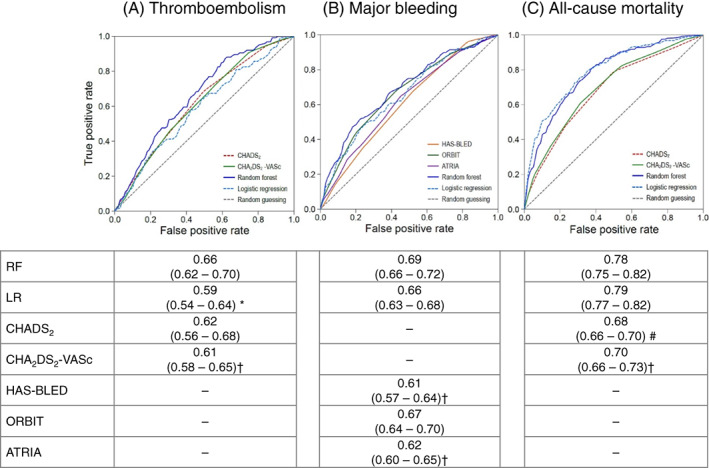
C‐statistics of the outcomes. The receiver operating characteristic curves for (A) thromboembolisms, (B) major bleeding, and (C) all‐cause mortality are shown in the upper figures. The C‐statistic and 95% confidence intervals are presented in the lower table. The C‐statistics of the RF were compared to that for the LR and clinical risk scores. RF: random forest, LR: stepwise logistic regression. The other abbreviations of the risk scores are shown in the supplementary file. Compared to RF: * <0.05, † < 0.01, # <0.001

Figure 1 compares the performance of the two models and various scoring systems in predicting the three types of outcomes. During a 2‐year follow‐up, 126 patients (1.6%) had thromboembolisms, 140 (1.8%) had major bleeding, and 195 (2.6%) died. The C‐statistic of the RF model for predicting thromboembolisms was 0.66 (95% CI 0.62–0.70), which was significantly higher than that of the LR model (0.59, *p* = .03) and CHA_2_DS_2_‐VASc score (0.61, *p* < .01) but was marginally higher than that of the CHADS_2_ score (0.62, *p* = .05). For major bleeding, the C‐statistic of the RF model (0.69, 95% CI 0.66–0.72) was comparable to that of the LR model (0.66, *p* = .07). The C‐statistic of the RF model outperformed the HAS‐BLED (0.61, *p* < .01) and ATRIA (0.62, *p* < .01), but not the ORBIT (0.67, *p* = .07). For the all‐cause mortality, no significant difference was observed in the C‐statistic between the RF model (0.78, 95% CI 0.75–0.82) and LR model (0.79, 95% CI 0.77–0.82). The discriminatory power of the RF model outperformed the CHADS_2_ score (0.68, *p* < .001) and CHA_2_DS_2_‐VASc score (0.70, *p* < .01) for predicting the 2‐year all‐cause mortality.

### 3.2 Permutation importance of the RF model

**FIGURE 2:**
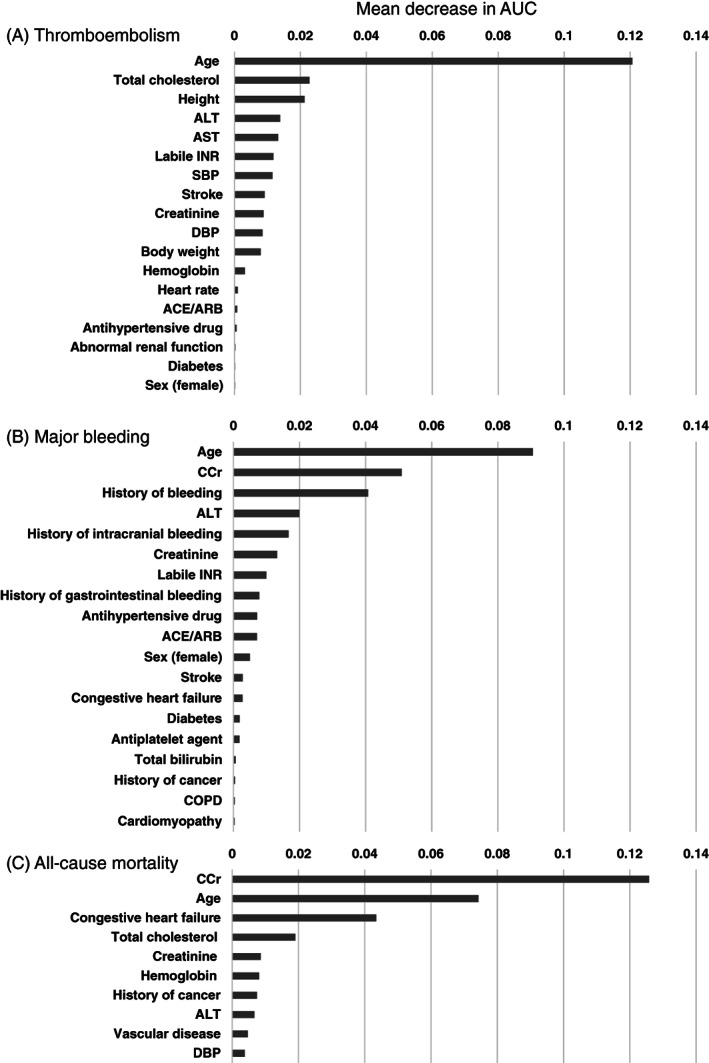
Permutation importance of the random forest model. Permutation importance for the classification of (A) thromboembolisms, (B) major bleeding, and (C) all‐cause mortality. The mean decrease in the AUC is a measure of the permutation importance. It shows how much a prediction made by the random forest model is degraded if a particular variable is shuffled (effectively removed). By inference, a variable with a larger decrease in the AUC must be contributing more to the model's predictive ability. AUC: area under the curve. The definitions of abnormal renal or hepatic function and other abbreviations are as in Table 1

The features in the order of the permutation importance of the RF model for predicting the three types of outcomes are shown in Figure 2. For thromboembolisms, in addition to previously known risk factors such as the age, systolic blood pressure, strokes, creatinine, and body weight, three new factors, the total cholesterol, height, and hepatic enzymes, were found to contribute to improving the model performance (Figure 2(A)). For major bleeding, the most predictive patient features in the order of a decreasing contribution included the age, creatinine clearance, and a history of any bleeding (Figure 2(B)). For the total mortality, creatinine clearance, age, and congestive heart failure were the three main features (Figure 2(C)). The permutation importance of risk factors in the LR model is shown in the Supplementary file (Figure S1).

### 3.3 Independent predictors of the stepwise LR model

**FIGURE 3:**
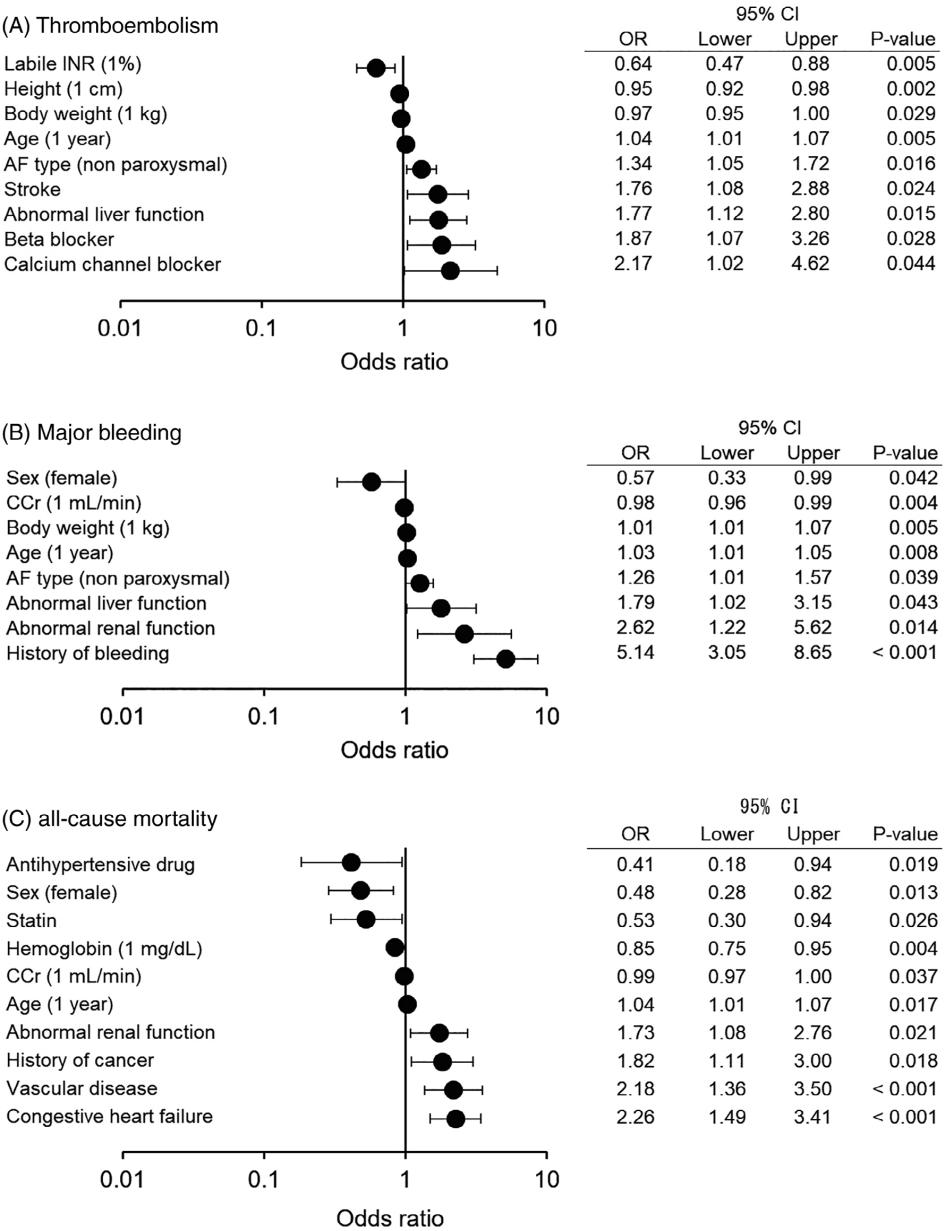
Independent predictors of the stepwise logistic regression model. The independent predictors and their odds ratio and 95% confidence interval of (A) thromboembolisms, (B) major bleeding, and (C) all‐cause mortality are shown. The definitions of abnormal renal or hepatic function and other abbreviations are as in Table 1

The independent predictors of the three types of outcomes found by the LR model are shown in Figure 3. Of the nine predictors found, the labile INR, height, body weight, age, and strokes were predictors common to the LR and RF models, but the type of AF and use of calcium channel blockers and beta‐blockers, were not picked up by the RF model (Figure 3(A)). For major bleeding, the independent predictors in the LR model not picked up by the RF model were the body weight and AF type (Figure 3(B)). For all‐cause mortality, the total cholesterol, ALT, and diastolic blood pressure were independent predictors not picked up by the RF model (Figure 3(C)).

### 3.4 Model calibration

**FIGURE 4:**
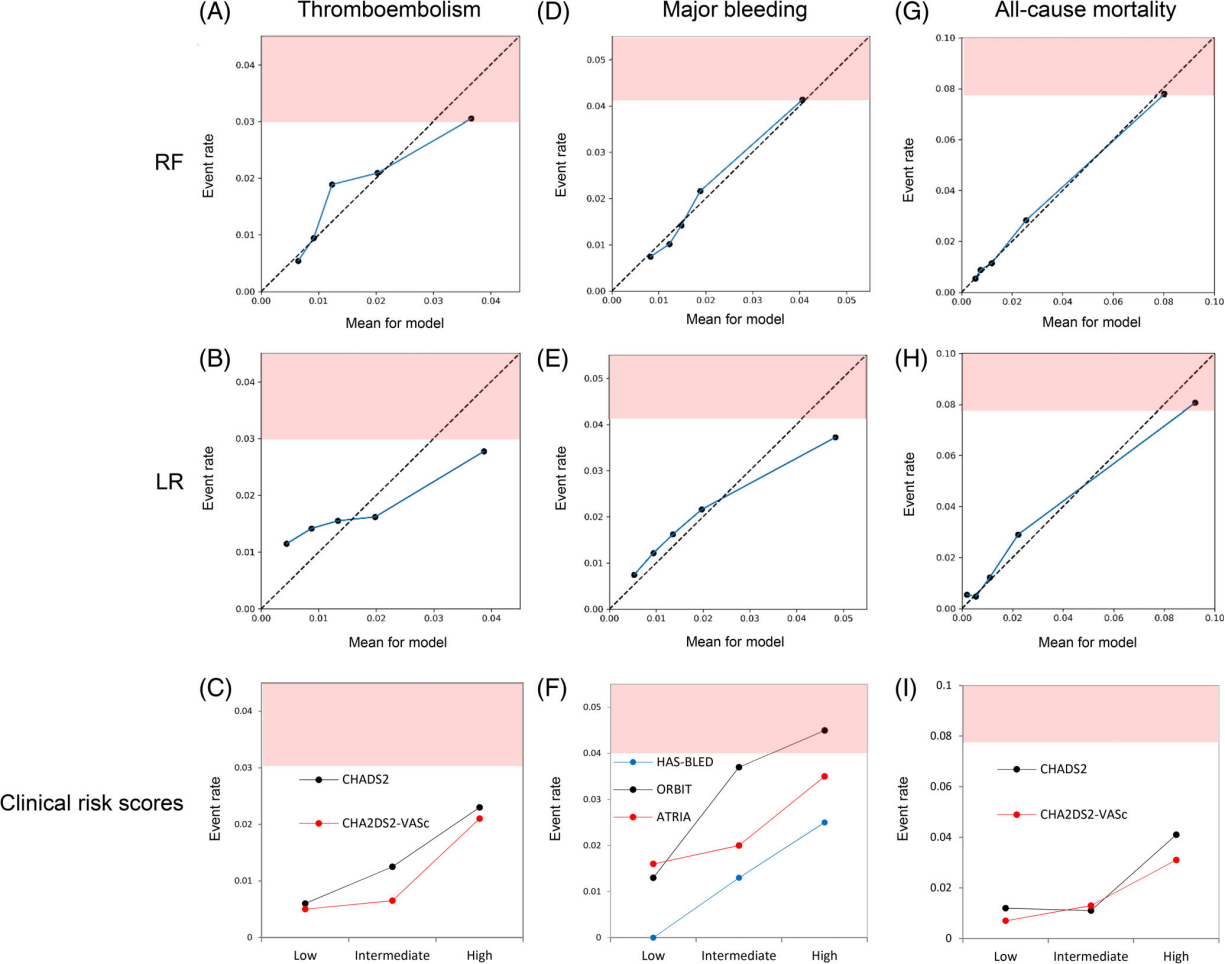
Calibration plots. The plots comparing the predicted event rates (horizontal axis) and observed event rates (vertical axis) for thromboembolisms (A, B, C), major bleeding (D, E, F), and all‐cause mortality (G, H, I) are shown. The blue line in the RF and LR indicates the trend for the calibration. When the intersect of the observed and expected event rates is below the dotted line, this indicates an overestimation of the event risks for that quintile. (C, F, I) The plots comparing the categorical score (horizontal axis) and observed event rates (vertical axis) are shown. The high‐risk event rate cutoff values were 3.0%, 4.1%, and 7.8% for thromboembolisms, major bleeding, and all‐cause mortality, respectively (red shaded area). The abbreviations and categorical grouping are shown in Table 1 and the Supplementary File (Appendix S1 and Table S1). RF: random forest, LR: stepwise logistic regression

In Figure 4, the continuous calibration plots for the RF and LR models, and categorical calibration of the risk scores are presented. With regard to thromboembolisms (Panels A ~ C), both the RF and LR models overestimated the actual observed event rate in the high‐risk (>3.0%) population, and the LR model underestimated the event rate in the lower risk population. The CHADS_2_ and CHA_2_DS_2_‐VASc scores underestimated the event rates for the high‐risk population. With regard to the major bleeding (Panels D ~ F), the calibration plot for the RF model approximated the observed event rate. The LR model overestimated the event rate in the high‐risk (>4.1%) population. The ORBIT score estimated the high‐risk population well. With regard to the all‐cause mortality (Panels G ~ I), the RF model showed good agreement between the estimated and observed event rates, while the LR model overestimated the event rate in the high‐risk (>7.8%) population. The CHADS_2_ and CHA_2_DS_2_‐VASc scores underestimated the event rates in the high‐risk population. The calibration plots for the continuous risk scores are presented in the Supplementary file (Figure S2).

### 3.5 Net reclassification improvement

The NRI for the outcomes between the models are presented in Supplemental Table S3. For thromboembolisms, the RF model more correctly identified events than the LR model and CHA_2_DS_2_‐VASc score. For major bleeding, the RF model showed no significant improvement in the discriminatory ability over the LR model or risk scores. For the all‐cause mortality, the RF model was no better than the LR model but was better than the CHADS_2_ and CHA_2_DS_2_‐VASc scores.

## 4 DISCUSSION

In this study, we compared the accuracy of the RF model, against the LR model and existing clinical risk scores for predicting three types of clinical outcomes of AF, namely, thromboembolisms, bleeding, and mortality, using a nationwide AF registry. The predictive performance of the RF model for thromboembolisms was modest but significantly outperformed the LR model and CHA_2_DS_2_‐VASc score. For major bleeding and all‐cause mortality, the predictive performance of the RF model was modest and comparable to the LR model, while it had a superior discrimination ability as compared to several risk scores. Our study suggests that the RF model performs as well as, or better than the LR model and conventional risk scores for predicting clinical outcomes in AF patients.

Despite the claims that ML models outperform conventional regression models in clinical medicine, few studies have compared the predictive performance between ML models and the LR model or existing risk scores in AF patients. Recently, Loring et al.^15^ examined the performance of three ML approaches (RF, gradient boosting, and neural networks) and the LR model in predicting strokes, major bleeding, and mortality, using two global AF registries (ORBIT‐AF and GARFIELD‐AF). The cross‐registry validation revealed that the LR model had a similar or better discrimination and calibration performance for these three outcomes compared to ML. They also reported the superiority of gradient boosting among the ML models. In our study, we showed that the discriminatory power of the RF model was highest for death (C‐statistic = 0.78) and lowest for thromboembolisms (C‐statistic = 0.66). These C‐statistic values were comparable to the abovementioned study by Loring et al., where the highest C‐statistic in the LR was for death (C‐statistic = 0.80 in ORBIT‐AF, 0.75 in GARFIELD‐AF) and the lowest C‐statistic was for strokes (C‐statistic = 0.67 in ORBIT‐AF, 0.66 in GARFIELD‐AF).^15^ In addition, the C‐statistic for major bleeding in the RF model in our study was 0.69, which was comparable to that of the LR model by Loring et al. (C‐statistic = 0.71 in ORBIT‐AF, 0.64 in GARFIELD‐AF). Our study examined the consistency of individual risk predictions between models to assess their usefulness in identifying patients at high risk. We found that the LR model underestimated the low risks and overestimated the high risks for thromboembolisms, probably due to overfitting. This pattern was repeated for major bleeding and mortality. The RF model, however, predicted major bleeding and mortality well. This observation was contrary to that of Loring et al., who found a well aligned calibration in the LR models. The cause of this difference is unknown, but many factors can play a role, such as the sample size, number of parameters considered, rate of missing data, patient race, drugs used (warfarin or direct oral anticoagulants), comorbidities, treatment or survival rate, number of censors,^14^ and tuning of the model hyperparameters.

ML models are often thought of as black boxes that take input and produce output. Interactions between the features and intermediate steps that affect output are poorly understood. The algorithm of the RF model is also a black box, but has the advantage of revealing factors (permutation importance) that contribute to improving the accuracy of the model and discovering complex interactions, even in high‐dimensional environments.^25^ At a high level, it works by randomly shuffling data for one feature at a time over the entire data set and calculating how much the performance metric of interest drops. Although the permutation importance is heuristic, it can correct the feature importance bias. To calculate the permutation importance, the number of permutations does not grow exponentially and is proportional to the number of the parameters. In this study, for example, the RF model selected the total cholesterol, height, hepatic enzymes, and labile INR for the risk estimation of thromboembolisms. Those variables have not been reported previously and are not considered in the LR model or existing risk scores. The LR model is often limited for data mining purposes because of interactions of multiple, nonlinear variables. The RF analysis, however, uses a nonparametric decision tree approach to overcome these issues. In other words, the risk factors selected by the RF model and not by the LR were such that the increase in their values was not related to a monotonic increase or decrease in the risk.

In this study, the RF model significantly improved the prediction of the outcomes when compared to the LR and standard clinical risk scores. Our approach used variables that are typically measured clinically. Although our understanding of the risk factors that regulate the risk of AF patients is based on clinical observation, there is limited information on the underlying mechanisms. Therefore, we supposed that the incorporation of the underlying mechanisms, such as the inflammatory cytokines,^29^ autonomic balance,^30^ atrial imaging parameters,^31^ or multiomics approaches^32^ may enable a more sophisticated risk stratification scheme. In addition to the above‐mentioned parameters, incorporation of population‐based risk factors such as the race/ethnicity, smoking, education, marital status, home ownership, and physical activity may further improve the predictive accuracy. Another advantage of RF is that we can describe the effect of each variable on an individualized prediction. Lack of interpretability of the novel features or patterns, however, raises some important questions for the clinician. We need to maximize both the accuracy and interpretability of the ML, but so far there is a trade‐off between the two. Currently, ML has limited clinical application for a risk assessment, but it could be utilized to personalize the risk assessment when programmed algorithms are implemented in electronic health records. We expect that ML will automatically collect variables and integrate all relevant clinical risk measurements to calculate the risk scores. Such a diagnostic support or computerized alerts may provide timely information that may improve the clinical decisions and potentially enhancing the therapeutic strategies. The prediction accuracy based on ML models depends on factors such as the data heterogeneity, ML choices, and feature selection algorithms. To test the clinical significance of our model, we need to validate them in multicenter datasets, clinical trials, and computational experiments.

### 4.1 Study limitations

This study had many strengths, including the large number of sites and patients studied and high quality of the clinical data collected through the registry, but had some limitations. The J‐Rhythm Registry was limited to cardiology practices that actively volunteered to participate in this nationwide registry and was not a randomized or blinded study. In this study, 86.5% of the patients were on anticoagulants, which may have confounded the models for the prediction of thromboembolisms and major bleeding. Additionally, no direct oral anticoagulants were used. This study was conducted with patients of Asian race only, therefore outcomes may differ in other races. Although the event rates for the three endpoints were very low (6.2%), we did not consider the class imbalance. To address this problem, we should apply a technique such as synthetic minority over‐sampling technique to achieve better classifier performance.^33^ In this study we used the RF model and did not employ support vector machine and neural network. The advantage of RF over the support vector machine and neural network is that RF works well for data analyses with a mixture of categorical and continuous values. In the future study, other types of ML algorithms should be tried.

## 5 CONCLUSIONS

Our study showed that the RF model performed as well as or better than the LR model or existing risk scoring schemes for predicting clinical outcomes. The RF model was also able to provide information on the relative importance of individual risk factors. The RF model has the potential to be implemented clinically and improve the decision making in patients with AF.

## CONFLICTS OF INTEREST

Dr. Watanabe received lecture fees from Daiichi‐Sankyo; Dr. Noyama has no COI; Dr. Kiyono received research funding from Kurabo Industries ltd.; Dr. Inoue received remuneration from Daiichi ‐Sankyo, Bayer Healthcare and Bristol‐Myers Squibb; Dr. Atarashi received lecture fees from Daiichi‐Sankyo; Dr. Okumura received research funding from Boehringer Ingelheim and Daiichi ‐Sankyo, and remuneration from Boehringer Ingelheim, Bayer Healthcare, Daiichi‐Sankyo, and Pfizer; Dr. Yamashita received research funding from Daiichi‐Sankyo, Bayer Healthcare and Bristol‐Myers Squibb, and remuneration from Boehringer Ingelheim, Daiichi ‐Sankyo, Bayer Healthcare, Pfizer, Bristol‐Myers Squibb, Ono Pharmaceutical and Toa Eiyo; Dr. Lip served as a consultant and speaker for BMS/Pfizer, Boehringer Ingelheim and Daiichi‐Sankyo; No fees are received personally. Dr. Kodani received lecture fees from Daiichi‐Sankyo; Dr. Origasa received lecture fees from Daiichi ‐Sankyo.

## Supporting information

**Table S1** Three category schemes
**Table S2**. Thromboembolism and major bleeding risk scores
**Table S3**. Net reclassification indices for random forest as compared to stepwise logistic regression and the existing risk scores.
**Figure S1**. Permutation importance of the stepwise logistic regression model.
**Figure S2**. Calibration plot for risk scores presented as continuous score.

## Data Availability

The data that support the findings of this study are available from the corresponding author upon reasonable request.
