# Supplementary material for: Comparison among random forest, logistic regression, and existing clinical risk scores for predicting outcomes in patients with atrial fibrillation: A report from the J‐RHYTHM registry
Source: Clin Cardiol. 2021 Jul 28;44(9):1305–15. doi: 10.1002/clc.23688 (PMC8427975; doi:10.1002/clc.23688)
Supplement: Supplementary file 1 — Table S1 Three category schemes Table S2. Thromboembolism and major bleeding risk scores Table S3. Net reclassification indices for random forest as compared to stepwise logistic regression and the existing risk scores. Figure S1. Permutation importance of the stepwise logistic regression model. Figure S2. Calibration plot for risk scores presented as continuous score. [file CLC-44-1305-s001.docx]

# **Supplementary file**

Watanabe E, et al.

**Appendix S1.**

Components of the CHADS_2_ score^1^ were defined by congestive heart failure, hypertension, age ≥75, diabetes, and stroke (2 points). Components of the CHA_2_DS_2_-VASc score ^2^ were defined by congestive heart failure, hypertension, age ≥75 (2 points), diabetes, strokes (2 points), vascular disease, age 65–74, and sex category (female). Components of the HAS-BLED score ^3^ were defined by hypertension, abnormal renal/liver function (1 point each), strokes, a bleeding history or predisposition, a labile international normalized ratio (INR) (therapeutic time in range (TTR)< 60%), elderly (>65 years), and use of drugs (antiplatelet agents, nonsteroidal anti-inflammatory drugs) or alcohol > 8 U/week (1 point each). Components of Outcomes Registry for Better Informed Treatment (ORBIT) bleeding risk score^4^ were defined by age >74 year, reduced hemoglobin/anemia (2 points), bleeding history (2 points), insufficient kidney function (eGFR <60 mL/min/1.73 m2), antiplatelet therapy. Components of ATRIA hemorrhage risk score^5^ were defined by anemia (3 points), severe renal disease (eGFR <30 mL/min/1.73 m2) (3 points), age >75 years (2 points), prior hemorrhage diagnosis, hypertension.

**Table S1. Three category schemes**

|  | CHADS_2_ | CHA_2_DS_2_-VASc | HAS-BLED | ORBIT | ATRIA |
| --- | --- | --- | --- | --- | --- |
| Low risk | 0 | 0 | 0 | 0 - 2 | 0 - 3 |
| Intermediate risk | 1 | 1 | 1 - 2 | 3 | 4 |
| High risk | >2 | >2 | >3 | 4 - 7 | 5 - 10 |

REFERENCES

1. Gage BF, Waterman AD, Shannon W, Boechler M, Rich MW and Radford MJ. Validation of clinical classification schemes for predicting stroke: results from the National Registry of Atrial Fibrillation. *JAMA*. 2001;285:2864-70.

2. Lip GY, Nieuwlaat R, Pisters R, Lane DA and Crijns HJ. Refining clinical risk stratification for predicting stroke and thromboembolism in atrial fibrillation using a novel risk factor-based approach: the Euro Heart Survey on atrial fibrillation. *Chest*. 2010;137:263-72.

3. Pisters R, Lane DA, Nieuwlaat R, de Vos CB, Crijns HJ and Lip GY. A novel user-friendly score (HAS-BLED) to assess 1-year risk of major bleeding in patients with atrial fibrillation: the Euro Heart Survey. *Chest*. 2010;138:1093-100.

4. O'Brien EC, Simon DN, Thomas LE, Hylek EM, Gersh BJ, Ansell JE, Kowey PR, Mahaffey KW, Chang P, Fonarow GC, Pencina MJ, Piccini JP and Peterson ED. The ORBIT bleeding score: a simple bedside score to assess bleeding risk in atrial fibrillation. *Eur Heart J*. 2015;36:3258-64.

5. Fang MC, Go AS, Chang Y, Borowsky LH, Pomernacki NK, Udaltsova N and Singer DE. A new risk scheme to predict warfarin-associated hemorrhage: The ATRIA (Anticoagulation and Risk Factors in Atrial Fibrillation) Study. *J Am Coll Cardiol*. 2011;58:395-401.

**Appendix S2.** Covariates used for the analysis.

**Continuous covariates:**

Age, years

Height (m)

Body weight (kg)

Heart rate (beat per min)

Systolic blood pressure (mmHg)

Diastolic blood pressure (mmHg)

Hemoglobin (g/dL)

Platelet (x 10^4^/uL)

Creatinine (mg/dL)

Creatinine clearance (mL/min)

Total cholesterol (mg/dL)

Time in therapeutic range of international normalized ratio of prothrombin time, %

**Categorical covariates:**

Male

Type of atrial fibrillation (paroxysmal, persistent, permanent)

Congestive heart failure

Hypertension

Age > 75 years

Diabetes

Previous stroke or transient ischemic attack

Coronary artery disease

Chronic obstructive pulmonary disease

Cardiomyopathy

Malignancy

Hepatitis

Abnormal renal function

Abnormal liver function

Alcohol abuse

Congenital heart disease

Hyperthyroidism

Previous bleeding

Class I antiarrhythmic drug

Class III antiarrhythmic drug

Beta-blocker

Calcium channel blocker

Digitalis

Angiotensin converting enzyme inhibitor

Angiotensin II type 1 receptor blocker

Statin

Warfarin

Antiplatelet agent

# **Table S2. Thromboembolism and major bleeding risk scores**

| Score | Points |
| --- | --- |
| CHADS_2_ score (points) | 1.7 ± 1.2 |
|  | 2 [1 – 2] |
| 0, n (%) | 1157 (15.6) |
| 1 | 2512 (33.9) |
| >2 | 3737 (50.5) |
| CHA_2_DS_2_-VASc score (points) | 2.8 ± 1.6 |
|  | 3 [2 – 4] |
| 0, n (%) | 602 (8.2) |
| 1 | 1194 (16.1) |
| >2 | 5610 (75.7) |
| HAS-BLED score (points) | 2.7 ± 1.2 |
|  | 3 [2 – 4] |
| 0, n (%) | 118 (1.5) |
| 1 – 2 | 3381 (45.7) |
| >3 | 3907 (52.8) |
| ORBIT score (points) | 1.5 ± 1.4 |
|  | 1 [1 – 2] |
| 0 – 2, n (%) | 5910 (79.8) |
| 3 | 768 (10.4) |
| 4-7 | 728 (9.8) |
| ATRIA (points) | 2.2 ± 2.1 |
|  | 1 [1 – 2] |
| 0 – 3, n (%) | 5801 (78.3) |
| 4 | 458 (6.2) |
| 5 – 10 | 1147 (15.5) |

The score abbreviations are shown in the Supplementary file. Each score was divided into 3 categories (low, intermediate, and high) based on the original literature. The data represent the number, frequency, means ± SD, or median [interquartile range].

# **Table S3. Net reclassification indices for random forest as compared to stepwise logistic regression and the existing risk scores.**

|  | Thromboembolism | Major bleeding | All-cause mortality |
| --- | --- | --- | --- |
| LR ^a)^ | 0.008  [0.002 – 0.014] * | 0.023  [-0.008 – 0.056] | 0.123  [-0.066 – 0.294] |
| CHADS_2_ ^b)^ | 0.093  [-0.012 – 0.198] |  | 0.290  [0.247 – 0.332] # |
| CHA_2_DS_2_-VASc ^b)^ | 0.121  [0.009 – 0.234] * |  | 0.338  [0.291 – 0.385] # |
| HAS-BLED ^b)^ |  | 0.070  [-0.005 – 0.147] |  |
| ORBIT ^b)^ |  | -0.048  [-0.152 – 0.055] |  |
| ATRIA ^b)^ |  | -0.029  [-0.159 – 0.101] |  |

Positive values reflect a more accurate classification and negative values reflect a less accurate classification. LR: stepwise logistic regression, the other abbreviations of risk scores are shown in the Supplementary file. a): continuous, b): categorical. Compared to the random forest: *: p < 0.05, #: p < 0.001.

**Figure S1. Permutation importance of the stepwise logistic regression model.**


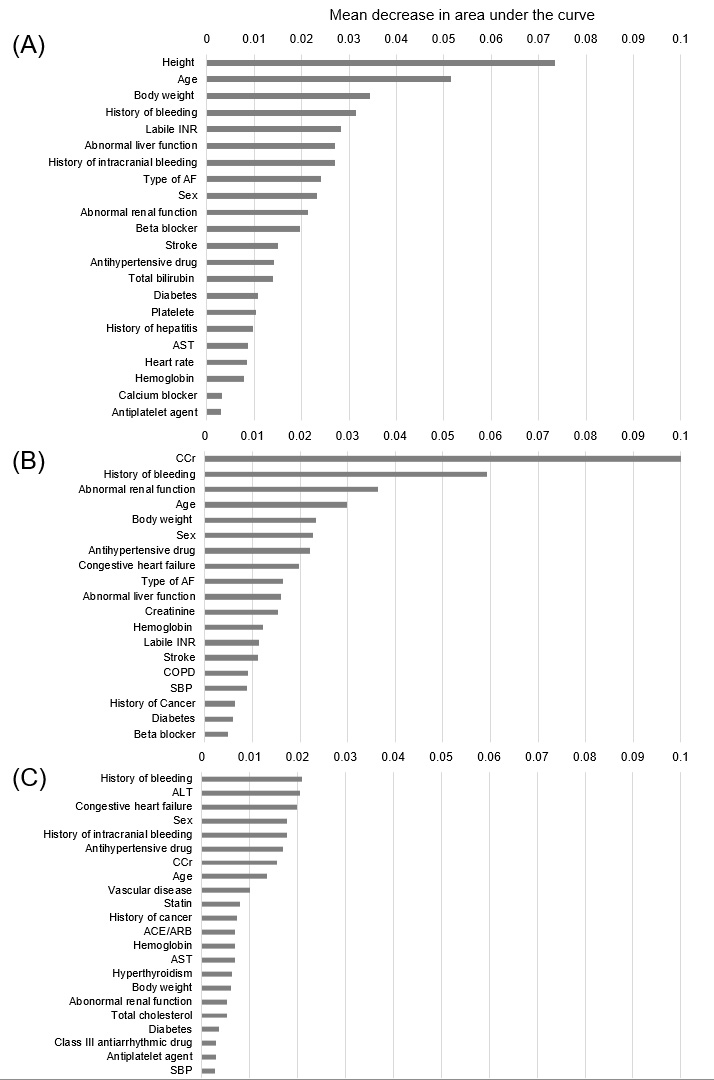


(A) Thromboembolism, (B) Major bleeding, and (C) All-cause mortality. The mean decrease in area under the curve denotes how much a feature contributes to the prediction made by the stepwise logistic model. Abbreviations were shown in Table 1 in the text.

**Figure S2. Calibration plot for risk scores presented as continuous score.**


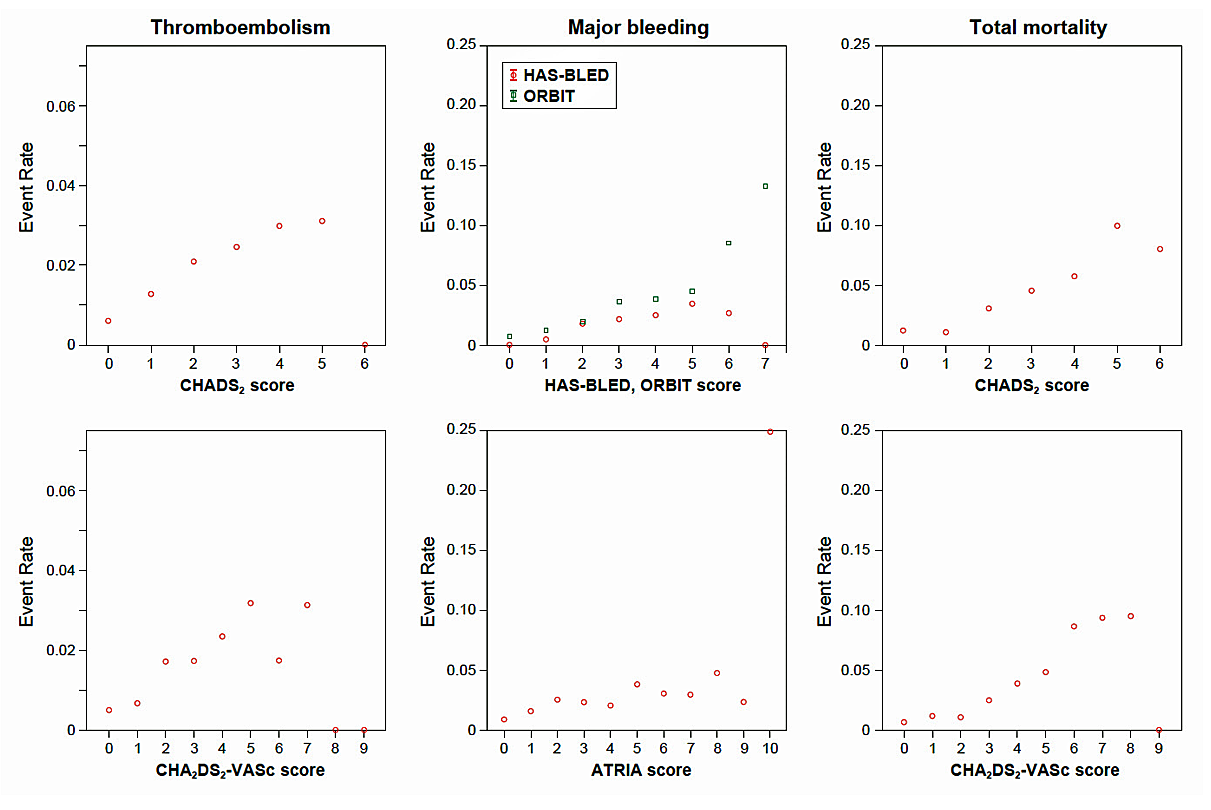


Abbreviations of the risk scores were shown in the 1^st^ paragraph of this document.
